# The Impact of Bone Mineral Biomarkers on Cardiac Dysfunction in Predialysis Chronic Kidney Disease Children

**DOI:** 10.1155/2021/4708452

**Published:** 2021-09-01

**Authors:** Safaa Ali, Mohamed Saber, Mohamed Kassem

**Affiliations:** Pediatric Department, Sohag University, Egypt

## Abstract

**Objective:**

To evaluate the association of bone mineral biomarkers of calcium, phosphorus metabolism, and 25-hydroxy vitamin D with diastolic dysfunction of the left ventricle and left ventricle mass in predialysis chronic kidney children. *Patients and Methods*. A cross-sectional observational study was conducted on 60 children with chronic kidney disease and treated by conservative treatment from October 2018 to September 2019 in the Pediatric Nephrology and Cardiology Department at our University Hospital.

**Results:**

The most common causes of CKD were congenital renal anomalies accounted for 22 (36.67%) of the studied cases. The mean age of children was 7.05 ± 2.74 years, and 32 (53.33%) were males. The children who had a normal diastolic function were 32 (53.33%), while those who had diastolic dysfunction were 28 (46.67%). There was a statistically significant in serum phosphorus (*p* value = 0.03), serum PTH (*p* value = 0.002), and hypertension (*p* value = 0.03). There was a statistically significant positive correlation between LVMI and iPTH level (*r* = 0.89, *p* ≤ 0.0001), 25(OH) cholecalciferol (*r* = −0.27, *p* = 0.04), serum Ca (*r* = −0.37, *p* = 0.004), and serum phosphorus (*r* = −0.45, *p* = 0.0003).

**Conclusion:**

Our results revealed that hyperparathyroidism, hyperphosphatemia, and hypertension were significantly associated with diastolic dysfunction while hypovitaminosis D was not significantly associated. Vitamin D deficiency was prevalent in all children with CKD. Biomarkers of mineral bone density were significantly associated with left ventricular hypertrophy and increased left ventricular mass index.

## 1. Introduction

Chronic kidney disease (CKD) is a major health problem worldwide in adults and pediatric population. The prevalence of pediatric CKD is 15-74.7 patients per 1 million children [1].

According to the kidney disease improving global outcome (KDIGO) guidelines, CKD is identified by the presence of kidney damage, either structural or functional or, by a decline in glomerular filtration rate (GFR) below 60 ml/min/1.73 m^2^ of body surface area for more than 3 months [2].

Cardiovascular disease (CVD) is the leading cause of morbidity and mortality among patients with chronic kidney disease [3]. CKD-associated cardiovascular morbidity (CVM) is more commonly reported in the form of diastolic cardiac dysfunction and left ventricular hypertrophy by echocardiography and used as an early marker of CVD. However, the systolic functions are preserved until the late stage [4, 5].

Many factors have been implicated in the etiology of the cardiac abnormalities observed in patients with CKD [6]. Recently, interest in the role of vitamin D as it relates to the cardiovascular system increased. Prior studies investigating adult patients with CKD have shown an association between vitamin D deficiency and the previously mentioned adverse cardiac changes, especially left ventricular hypertrophy [7].

Vitamin D may have cardiovascular effects through several mechanisms. 25(OH)D is a known regulator of the renin-angiotensin-aldosterone system (RAAS), and treatment with calcitriol leads to renin suppression in experimental studies. Vitamin D receptor is present on vascular smooth muscle cells, and calcitriol can affect the proliferation of these cells [8].

The current study was conducted to assess the cardiovascular changes in children with CKD and detect the association of these changes with markers of calcium and phosphorus metabolism, including 25-hydroxy vitamin D.

## 2. Patient and Method

The study was a prospective, observational cohort study of pediatric CKD over one year from October 2018 to September 2019. The study was done in the Pediatric Nephrology and Cardiology Department of Sohag University Hospital. The study included 60 children with chronic kidney disease and treated by conservative treatment. The cases were subsequently divided into two groups according to diastolic dysfunction.

*Inclusion Criteria:* All children (2–18 years) with CKD and treated by conservative (predialysis) treatment were included in this study.

*Exclusion Criteria:* Patients with primary cardiac disease (e.g., congenital heart disease, congenital anomalies of coronaries or rheumatic heart disease, and cardiomyopathy).

*Ethical considerations:* Must be complete.

The study was approved by the Institutional Ethics Committee. Informed written consent was obtained from either parent of every included patient.

All patients were subjected to the following:

(1) Full history taking includes demographic data, age, sex, child's previous growth and development, and history about the primary cause of CKD
(2) Clinical examination including the following:
  - Anthropometric measurements for assessment of nutritional and developmental status included: weight and height
  - Body mass index (BMI) percentile—(<5%, 5%–85%, and ≥85%). Height and body mass index (BMI; kg/m^2^ of height) percentiles by age and sex were calculated from tables provided by the Centers for Disease Control and Prevention

BMI was calculated by the following formula: *BMI* = *Weight* (*kg*)/(*height* [*m*])

(c) Vital signs, especially arterial blood pressure

(3) Routine laboratory investigations including the following:
  - Complete blood count (CBC) by an automated analyzer

Anemia was defined as a mean hemoglobin value <11 g/dl

(b) Blood urea, blood urea nitrogen, and serum creatinine
(c) Serum albumin and serum electrolytes (ionized calcium, potassium, and phosphorus)
(d) A serum level of 25(OH) vitamin D was measured in a venous blood sample in all included patients. We considered vitamin D “deficiency” if 25(OH) D value below 20 ng/ml and “insufficiency” if 25(OH) D value between 20 and 30 ng/ml. Vitamin D deficiency was further classified as severe deficiency < 10 ng/ml and moderate deficiency 10–20 ng/ml
(e) The glomerular filtration rate (GFR) was estimated using the Schwartz formula [9]

(4) Doppler echocardiography, tissue Doppler imaging, and *M*-mode echocardiography:

Echocardiography was performed in all patients using GE Vivid 9 ((GE Medical System, Horten, Norway, with a 3.5 MHz multifrequency transducer) to evaluate cardiac structure and function by senior echocardiographer.

- Left ventricular mass index (LVMI) is a parameter used in echocardiography and cardiac MRI which is used to evaluate LV hypertrophy. Left ventricular mass (LVM) was calculated using the measurements obtained by two-dimensional *M*-mode echocardiography according to the American Society of Echocardiography (ASE) criteria. Indexed LVM (LVMI) was calculated using the following equations: LVMI = LVM (left ventricular mass)/body surface area or dividing the LVM by height raised to a power of 2.7 (g/m^2.7^) using the formula devised by De Simone et al. [10].
- Diastolic function was assessed by both Doppler echocardiography and tissue Doppler imaging. Early mitral inflow velocity (*E*) and late mitral inflow velocity (A) were measured by Doppler echocardiography. Tissue velocity imaging (TVI) was done in the four-chamber view with the mitral annular planes perpendicular to the ultrasound beam. A 5 mm pulsed TD sample volume was placed at the septal and lateral aspects of mitral annulus as well as at the lateral aspect of tricuspid annulus. Early diastolic (*e*′) (early diastolic filling velocity) and late peak diastolic myocardial (*a*′) peak mitral annular velocities were measured at the medial and lateral mitral annulus using tissue Doppler. The *e*′/*a*′ ratio at both annuli was calculated. The peak early mitral annular velocity (*E*′) was computed as the average of the velocities at the medial and lateral annuli. Using these measurements, the ratio of Doppler-derived peak early mitral inflow velocity to tissue Doppler-derived peak early mitral annular velocity (*E*/*E*′ ratio) was calculated. Diastolic dysfunction was graded as follows: grade 0, no diastolic dysfunction; grade 1 (impaired relaxation), *E*/*E*′ ≤ 8; grade 2 (pseudonormal), *E*/*E*′ 9–12; and grade 3 (restrictive grade) *E*/*E*′ ≥ 13 [11].
- Myocardial performance index (MPI), or Tei index, is a Doppler echocardiographic parameter defined as the sum of the isovolumic contraction and relaxation times divided by the ejection time. It is considered a reliable parameter for evaluating left ventricular systolic and diastolic dysfunction

## 3. Statistical Analysis

The collected data were coded, processed, and analyzed using the SPSS (Statistical Package for Social Sciences) version 22 for Windows® (IBM SPSS Inc, Chicago, IL, USA). Data were tested for normal distribution using the Shapiro Walk test. Qualitative data were represented as frequencies and relative percentages. Chi-square test is used (*χ*^2^) to calculate the difference between two or more groups of qualitative variables. Quantitative data were expressed as mean ± SD (standard deviation). Independent samples *t*-test was used to compare between two independent groups of normally distributed variables (parametric data). *p* value < 0.05 was considered significant.

## 4. Results

A prospective observational study was done over 12 months, from October 2019 to September 2020. The study was conducted on 60 patients with chronic kidney disease (stage 3 or 4), and their age range from 2 to 13 years with a mean value of 7.05 ± 2.74 years. They were 32 (53.33%) males and 28 (46.67%) females. The mean weight was 19.43 ± 7.01 kilograms ranging from 8 : 39 kilograms. The mean height was 111.57 ± 18.06 cm ranging from 79 : 148 cm. The mean body mass index was 14.95 ± 1.30 kg/m^2^ ranging from 12.8 : 17.8 kg/m^2^. 40 (66.67%) of the studied patient was healthy, and 20 (33.33%) of them was underweight (Table 1).

The most common causes of CKD were congenital renal anomalies 22 (36.67%) of studied patients. Twenty-one (35.00%) of the patients had focal segmental GS. We reported that there were 20 (33.33%) of the children who had stage 3 (A and B) and 40 (66.67%) had stage 4 (Table 2).

Thirty-two (53.33%) of the studied patients had normal diastolic function (group A) while 28 (46.67%) had grade II diastolic dysfunction (group B).

Diastolic dysfunction observed in the children with CKD was evidenced by a lower *E*′ and a higher *E*/*E*′ ratio.

Comparing group A with group B, there was statistically significant difference in mean *E*/*E*′ (*p* value < 0.0001), mitral *E* velocity (*p* value = 0.009), lateral mitral annulus *E*′ (*p* value < 0.0001), septal mitral annulus *E*′ (*p* value < 0.0001); meanwhile, there was no statistically significant difference in mitral valve *E*/*A* ratio (*p* value = 0.89) (Table 3).

Comparing group A with group B as regards the demographic data and anthropometric measurements revealed that there was no statistically significant difference in age (*p* value = 0.58), gender (*p* value = 0.58), consanguinity (*p* value = 0.18), weight (*p* value = 0.93), height (*p* value = 0.77), and body mass index (*p* value = 0.89) Table 4.

Comparing group A with group B, there was no statistically significant difference in the causes of CKD (*p* value = 0.997) and GFR (*p* value = 0.2), while there is statistically significant difference in stages of CKD (*p* value = 0.03). There were 24 of the studied patients in group B who were hypertensive (*p* value = 0.002) (Table 5).

As regards laboratory finding in group A and group B, there was no statistically significant difference in WBCs (*p* value = 0.38), Hb (*p* value = 0.06), PLTs (*p* value = 0.2), and serum creatinine (*p* value = 0.14) (Table 6).

**Figure 1:**
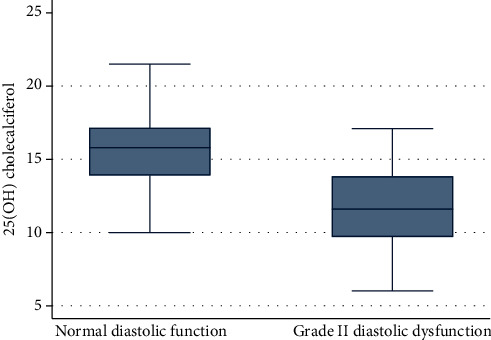
The relation between diastolic dysfunction and 25(OH) cholecalciferol.

**Figure 2:**
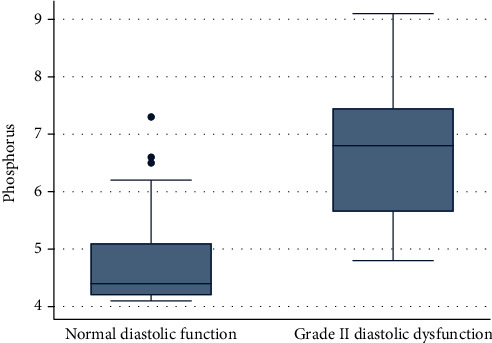
The relation between diastolic dysfunction and phosphorus.

**Figure 3:**
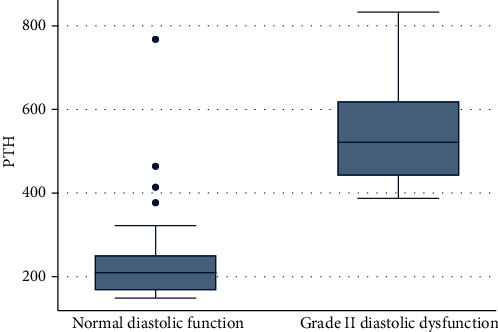
Relation between diastolic dysfunction and parathyroid hormone.

Vitamin D deficiency was prevalent in children with CKD. The mean 25-hydroxy vitamin D level in a group A was 15.70 ± 3.02 while in a group B was 11.74 ± 2.68 (*p* value < 0.0001). When the cases were subdivided into two groups using a cutoff value of 15 ng/ml for the mean 25-hydroxy vitamin D level, we found the higher proportion in group B was 25 (89.29%) while in group A was 14 (43.75%); this was found to be statistically significant (*p* value < 0.0001). The mean level of serum phosphorus in group A was 4.78 ± 0.85 while in group B was 6.70 ± 1.29; this was found to be statistically significant (*p* value < 0.0001). The mean serum levels of intact PTH in group A was 240.1 ± 124.44 while in group B was 532.91 ± 120.54; this was found to be statistically significant (*p* value < 0.0001) (Figures 1–3 and Tables 6 and 7).

Multivariate logistic regression of factors that may affect diastolic dysfunction revealed that there was no statistically significant in stages of CKD (*p* value = 0.07), serum Ca (*p* value = 0.82), and serum 25(OH) cholecalciferol (*p* value = 0.59) while there was statistical significance in serum phosphorus (*p* value = 0.03), serum PTH (*p* value = 0.002), and hypertension (*p* value = 0.03) (Table 8).

By correlation coefficient test (*r*), there was statistically significant positive correlation between LVMI and iPTH level (*r* = 0.89, *p* ≤ 0.0001), 25(OH) cholecalciferol (*r* = −0.27, *p* = 0.04), serum Ca (*r* = −0.37, *p* = 0.004), and serum phosphorus (*r* = −0.45, *p* = 0.0003) (Table 9).

## 5. Discussion

Some studies have suggested the progression of CKD, and the associated cardiovascular diseases may be linked to vitamin D insufficiency or deficiency [18, 19]. However, our study demonstrated that there was no correlation with vitamin D deficiency while the serum PTH levels significantly correlated with diastolic dysfunction of the studied children as evidenced by their echocardiographic data, *E*/*E*′ ratio.

The number of cases in this cohort study was 60 patients with CKD (stage 3 or 4); they were 32 (53.33%) males and 28 (46.67%) females, the median age of children was 7 (2 : 13), the median weight was 18 (8 : 39) while the median height was 113.5 (79 : 148) when compared to EL-Gamasy et al.'s [12] study, where the number of cases was 86 with CKD (stage 4 or 5), they were 48 (55.8%) males and 38 (44.2%) females, their age of them ranged from 10 to 18 years with a mean value of 13.7 ± 3.9 years, and the number of control was 40.

In Patange et al. [13], the number of cases was 34 children with CKD, 20 were receiving dialysis, and 14 were not receiving any form of dialysis for the management of CKD, 19 (55.9%) males, and 15 (44.1%) females; their mean age 14.7 ± 2.6.

Our results had reported that the most common causes of CKD were congenital renal anomalies 22 (36.67%); incomparable with EL-Gamasy et al. [12], the most common causes were difficult to treat nephrotic syndrome 44 (51.2%).

We had reported that the number of cases that had normal diastolic function was 32 (53.33%), while those had diastolic dysfunction were 28 (46.67%), in concordance with EL-Gamasy et al.'s study [12].

In the present study, the anthropometric measurements in group A (normal diastolic function) and group B (grade II diastolic dysfunction) were no statistically significant difference in age (*p* value = 0.58), gender (*p* value = 0.58), consanguinity (*p* value = 0.18), weight (*p* value = 0.93), height (*p* value = 0.77), and body mass index (*p* value = 0.89) which was in agreement with the results found in EL-Gamasy et al.'s [12] study where there was no statistically significant difference in age (*p* value = 0.46) and gender (*p* value = 0.29). In Patange et al. [13], the same results were found where there was no statistically significant difference in age, gender, and BMI (*p* value not significant).

We observed that there was no statistically significant difference between two groups in the causes of CKD (*p* value = 0.997) and GFR (*p* value = 0.2), while there statistically significant difference in stages of CKD (*p* value = 0.03) which was in agreement with EL-Gamasy et al.'s [12] study where there was no statistically significant difference in causes (*p* value = 0.09) and GFR (*p* value > 0.05).

The results of the present study revealed that 39 (65%) of the studied patients had hypertension, 24 (85.71%) had grade II diastolic dysfunction, 15 (46.88%) had normal diastolic function (*p* value = 0.002), which was in agreement with EL-Gamasy et al.'s [12] study, 53 (61.6%) of the studied patients were hypertensive (*p* value < 0.001), and the same results were found in Patange et al. [13] study where there were 28 (82.4%) of the studied patient who were hypertensive (*p* value ˂ 0.05). Hypertension is an independent common risk factor for cardiovascular diseases in children with CKD.

The current study had shown that the laboratory finding in group A and group B was no statistically significant difference in WBCs (*p* value = 0.38), Hb (*p* value = 0.06), PLTs (*p* value = 0.2), and serum creatinine (*p* value = 0.14) that was not in agreement with EL-Gamasy et al. [12] that had been reported that there was no statistically significant difference in serum creatinine (*p* value > 0.05), but there was a statistically significant difference in Hb (*p* value = 0.0002).

Our results revealed the mean level of serum phosphorus in group A was 4.78 ± 0.85 while in group B was 6.70 ± 1.29 (*p* value < 0.0001).

Hyperphosphatemia was correlated with indices of diastolic dysfunction as evidenced by the echocardiographic data, mean *E*/*E*′ (*r* = 0.61, *p* value < 0.0001), lateral mitral annulus *E*′ (*r* = −0.55, *p* value < 0.0001), and septal mitral annulus *E*′ (*r* = −0.49, *p* value = 0.00001). We demonstrated that hyperphosphatemia was considered a significant risk factor for the development of diastolic dysfunction in cases with CKD.

This was in agreement with EL-Gamasy et al.'s [12] study which had reported a mean level of serum phosphate of 7.3 ± 2.4 mg/dl and 5.5 ± 1.4 mg/dl in cases with and without diastolic dysfunction.

Also, our study agreed with Mahdi et al. [14] study who emphasized that hyperphosphatemia that promotes loss of minerals from bone can also promote metastatic or vascular calcification.

Galetta et al. [15] study also demonstrated a statistically significant positive correlation between hyperphosphatemia and increased CVD in their uremic patients on maintenance hemodialysis.

Furthermore, our results show that the mean serum levels of intact PTH in group A was 240.1 ± 124.44 while in group B was 532.91 ± 120.54; this was found to be statistically significant (*p* value < 0.0001). Hyperparathyroidism was correlated with indices of diastolic dysfunction as evidenced by the echocardiographic data, mean *E*/*E*′ (*r* = 0.63, *p* value < 0.0001), lateral mitral annulus *E*′ (*r* = −0.38, *p* value = 0.003), and septal mitral annulus *E*′ (*r* = −0.36, *p* value = 0.004). Hence, we reported that hyperparathyroidism was a significant risk factor for the development of diastolic dysfunction in cases with CKD.

Our results were in agreement with Patange et al. [13] who reported that in their study of 34 pediatric patients with CKD, the serum PTH levels correlated with diastolic dysfunction of their studied children as evidenced by their echocardiographic data, *E*/*E*′ ratio (*r* = 0.6, *p* < 0.05) and *E*′ (*r* = −0.6, *p* value < 0.05).

Strózecki et al.'s study was in accordance to our results which had reported increased serum phosphate level in CKD which is predisposing to the development of secondary hyperparathyroidism and to elevated Ca xPo4 leading to extravascular metastatic calcification. Both conditions involved in cardiac morbidity and mortality in CKD [16].

Hypovitaminosis D was found in both group A and group B. The mean 25-hydroxy vitamin D level in group A was 15.70 ± 3.02 while in group B was 11.74 ± 2.68, respectively. Notably, the interrelations among variables and multivariate logistic regression of factors revealed that hyperphosphatemia, hyperparathyroidism, and hypertension were the most risk factors to have a serious hazardous impact on left ventricular function especially diastolic dysfunction.

The results of our study were in accordance to which reported by EL-Gamasy et al. [12] who demonstrated that there was no statistically significant association between vitamin D deficiency and diastolic dysfunction in CKD patients (*p* = 0.3), which also was following the report by Pandit et al. [17].

Our results were not in agreement with Patange et al.'s [13] study which emphasized that hyperparathyroidism was linked to diastolic dysfunction suggesting the increased PTH level observed in children with CKD was secondary to chronic vitamin D deficiency, thus supposed that the diastolic dysfunction was linked to vitamin D deficiency as well.

Our study encompassed the correlation coefficient test (*r*) which confirmed a statistically significant positive correlation between LVMI and iPTH level (*r* = 0.89, *p* ≤ 0.0001) that was in agreement with EL-Gamasy et al.'s [12] study who reported that there was a statistically significant between iPTH and LVMI (*r* = 0.47, *p* < 0.05). Furthermore, Al-Hilali et al.'s [18] study found a positive correlation between iPTH and LVMI in their study on their patients on hemodialysis.

We demonstrated a positive correlation between 25(OH) cholecalciferol and LVMI (*r* = −0.27, *p* value = 0.04); this was in agreement with Patange et al.'s [13] study of 34 children with CKD that showed that LVMI inversely correlated with vitamin D, and it was also statistically significant (*r* = −0.5; *p* < 0.05). Meanwhile, EL-Gamasy et al.'s [12] study had not been reported any significant correlation between 25(OH) cholecalciferol and LVMI (*r* = −0.02, *p* > 0.05).

## 6. Study Limitation

Limitations of the present study include small cohort pediatric patients. The tissue Doppler indicators of diastolic dysfunction were not normalized to age-independent *z*-score. Another obvious limitation of our study is the cross-sectional design of our analysis, not a case-control one.

## 7. Conclusion

Our results emphasized that hyperparathyroidism, hyperphosphatemia, and hypertension are significantly associated with diastolic dysfunction while hypovitaminosis D was not significantly associated. Vitamin D deficiency was prevalent in all children with CKD. Biomarkers of mineral bone density were significantly associated with left ventricular hypertrophy and increased left ventricular mass index.

## Figures and Tables

**Table 1:** Distribution of the studied patients according to demographic data and anthropometric measures (*N* = 60).

Variable	No. (%)
*Age/years*	
Mean ± SD	7.05 ± 2.74
Median (range)	7 (2 : 13)

*Gender*	
Female	28 (46.67%)
Male	32 (53.33%)

*Weight*	
Mean ± SD	19.43 ± 7.01
Median (range)	18 (8 : 39)

*Height*	
Mean ± SD	111.57 ± 18.06
Median (range)	113.5 (79 : 148)

*BMI*	
Mean ± SD	14.95 ± 1.30
Median (range)	15.05 (12.8 : 17.8)

*BMI*	
Underweight	20 (33.33%)
Healthy	40 (66.67%)

*Consanguinity*	
Negative	27 (45.00%)
Positive	33 (55.00%)

**Table 2:** Causes and stages of CKD of studied population.

Variable	No. (%)
*Causes of CKD*	
Congenital renal anomalies	22 (36.7%)
Focal segmental GS	21 (35.00%)
Chronic glomerulonephritis	11 (18.33%)
Chronic interstitial nephritis	6 (10.00%)

*Stages of CDK*	
Stage 3 A	18 (30%)
Stage 3 B	2 (3.33%)
Stage 4	40 (66.67%)

**Table 3:** The relation between echo finding and diastolic dysfunction.

Variable	Group A *N* = 32	Group B *N* = 28	*p* value
*MeanE*/*E*′			
Mean ± SD	8.08 ± 1.31	11.41 ± 0.58	<0.0001
Median (range)	7.9 (6.5 : 9.9)	11.25 (10.9 : 12.4)	

*MitralEvelocity*			
Mean ± SD	1.01 ± 0.14	1.11 ± 0.14	0.009
Median (range)	0.98 (0.77 : 1.19)	1.15 (0.92 : 1.32)	

*Lateral mitral annulusE*′			
Mean ± SD	0.13 ± 0.02	0.11 ± 0.02	<0.0001
Median (range)	0.13 (0.1 : 0.16)	0.1 (0.09 : 0.15)	

*Septal mitral annulusE*′			
Mean ± SD	0.12 ± 0.02	0.09 ± 0.02	0.0001
Median (range)	0.12 (0.08 : 0.14)	0.09 (0.06 : 0.12)	

*MVE*/*Aratio*			
Mean ± SD	1.53 ± 0.26	1.53 ± 0.25	0.87
Median (range)	1.54 (1.3 : 2.03)	1.46 (1.16 : 2)	

**Table 4:** The relation between diastolic dysfunction, patient characteristic, and anthropometric measurements of children with CKD.

Variable	Group A *N* = 32	Group B *N* = 28	*p* value
*Age/years*			
Mean ± SD	7.23 ± 2.58	6.89 ± 2.94	0.58
Median (range)	7 (3 : 12)	7 (2 : 13)	

*Gender*			
Female	16 (50.00%)	12 (42.86%)	0.58
Male	16 (50.00%)	16 (57.14%)	

*Consanguinity*			
Negative	17 (53.13%)	10 (35.71%)	0.18
Positive	15 (46.88%)	18 (64.29%)	

*Weight*			
Mean ± SD	19.42 ± 6.90	19.43 ± 7.26	0.93
Median (range)	18 (10 : 33)	18.5 (8 : 39)	

*Height*			
Mean ± SD	111.78 ± 17.98	111.32 ± 18.48	0.77
Median (range)	112 (84 : 141)	113.5 (79 : 148)	

*BMI*			
Mean ± SD	14.93 ± 1.31	14.98 ± 1.32	0.89
Median (range)	14.75 (12.9 : 17.6)	15.2 (12.8 : 17.8)	

*BMI group*			
Healthy	10 (31.25%)	10 (35.71%)	0.71
Underweight	22 (68.75%)	18 (64.29%)	

**Table 5:** The relation between diastolic function, causes, stages, blood pressure, and GFR.

Variable	Group A *N* = 32	Group B *N* = 28	*p* value
Congenital renal anomaly	12 (37.50%)	10 (35.71%)	0.997
Focal segmental GN	11 (34.38%)	10 (35.71%)	
Chronic glomerulonephritis	6 (18.75%)	5 (17.86%)	
Chronic interstitial nephritis	3 (9.38%)	3 (10.71%)	

*Blood pressure*			
Normotensive	17 (53.13%)	4 (14.29%)	0.002
Hypertensive	15 (46.88%)	24 (85.71%)	

*GFR*			
Mean ± SD	25.03 ± 11.19	28.52 ± 9.34	0.20
Median (range)	20.2 (11 : 55)	30.9 (11.5 : 47.5)	

*Stages of CKD*			
Stage 3 A	5 (15.63%)	13 (46.43%)	0.03
Stage 3 B	1 (3.13%)	1 (3.57%)	
Stage 4	26 (81.25%)	14 (50.00%)	

*Stages of CKD*			
Stage 3 A/B	6 (18.75%)	14 (50.00%)	0.01
Stage 4	26 (81.25%)	14 (50.00%)	

**Table 6:** The relation between diastolic dysfunction and lab investigation of children with CKD.

Variable	Group A *N* = 32	Group B *N* = 28	*p* value
*WBCs*			
Mean ± SD	8.18 ± 1.29	8.59 ± 2.20	0.38
Median (range)	8 (5.8 : 11.6)	8.05 (5.3 : 14.9)	

*HB*			
Mean ± SD	10.01 ± 1.12	10.56 ± 1.08	0.06
Median (range)	10.05 (7.4 : 13)	10.5 (8.6 : 13.4)	

*Anemia (HB* < 11 *gm*/*dl*)			
No	8 (25.00%)	7 (25.00%)	1.00
Yes	24 (75.00%)	21 (75.00%)	

*HCT*			
Mean ± SD	29.20 ± 5.77	30.75 ± 4.27	0.24
Median (range)	30.95 (19 : 38.4)	31.75 (19.5 : 36.8)	

*MCV*			
Mean ± SD	75.41 ± 6.60	75.95 ± 4.64	0.72
Median (range)	75.25 (65 : 96.6)	77 (63 : 88.6)	

*PLTs*			
Mean ± SD	294.63 ± 82.72	276.79 ± 102.27	0.20
Median (range)	296 (156 : 450)	274 (134 : 614)	

*Serum creatinine*			
Mean ± SD	2.59 ± 0.92	2.24 ± 0.86	0.14
Median (range)	2.5 (1.1 : 4.4)	1.9 (1.1 : 4.7)	

*Ca*			
Mean ± SD	8.29 ± 0.61	7.55 ± 0.63	<0.0001
Median (range)	8.35 (6.8 : 9.5)	7.35 (6.7 : 8.8)	

*Phosphorus*			
Mean ± SD	4.78 ± 0.85	6.70 ± 1.29	<0.0001
Median (range)	4.4 (4.1 : 7.3)	6.8 (4.8 : 9.1)	

*PTH*			
Mean ± SD	240.1 ± 124.44	532.91 ± 120.54	<0.0001
Median (range)	210.05 (148.7 : 767.5)	521 (387.5 : 832.5)	

*25(OH) cholecalciferol*			
Mean ± SD	15.70 ± 3.02	11.74 ± 2.68	<0.0001
Median (range)	15.8 (10 : 21.5)	11.6 (6.02 : 17.1)	

*25(OH) cholecalciferol*			
>15 ng	18 (56.25%)	3 (10.71%)	<0.0001
<15 ng	14 (43.75%)	25 (89.29%)	

**Table 7:** The relation between indices of diastolic function, serum Ca, serum phosphorus, serum PTH, and serum 25(OH) cholecalciferol.

Cardiovascular outcomes of interest	Significant predictors at univariate analysis	Correlation coefficient (*r*)	*p* value
Mean *E*/*E*′	PTH	0.63	<0.0001
	25(OH)cholecalciferol	-0.48	0.0001
	Ca	-0.53	<0.0001
	Phosphorus	0.61	<0.0001

Lateral mitral annulus *E*′	PTH	-0.38	0.003
	25(OH)cholecalciferol	0.18	0.018
	Ca	0.55	<0.0001
	Phosphorus	-0.55	<0.0001

Septal mitral annulus *E*′	PTH	-0.36	0.004
	25(OH)cholecalciferol	0.23	0.07
	Ca	0.43	0.0006
	Phosphorus	-0.49	0.00001

**Table 8:** Multivariate logistic regression of factors that may affect diastolic dysfunction.

Variable	Odds ratio (95% confidence interval	*p* value
*Stages of CKD*		
Stage 3 A/B	1	
Stage 4/5	0.14 (0.02 : 1.13)	0.07

*Ca*	0.70 (0.04 : 13.5)	0.82
*Phosphorus*	7.26 (1.22 : 43.34)	0.03
*PTH*	1.02 (1.01 : 1.03)	0.002
*25(OH) cholecalciferol*		
>15 ng	1	
<15 ng	1.74 (0.23 : 13.32)	0.59
*Hypertension*	19.79 (1.46 : 267.44)	0.03

**Table 9:** The relation between left ventricular mass index, serum Ca, serum phosphorus, serum PTH, and 25(OH) cholecalciferol.

Cardiovascular outcomes of interest	Significant predictors at univariate analysis	Correlation coefficient (*r*)	*p* value
Left ventricular mass index	PTH	0.89	<0.0001
	25(OH)cholecalciferol	-0.27	0.04
	Ca	-0.37	0.004
	Phosphorus	0.45	0.0003

## Data Availability

The data will be available on request through a data access committee, institutional review board, or the authors themselves.
